# Supplementary material for: In-cell structure and variability of pyrenoid Rubisco
Source: Nat Commun. 2025 Aug 20;16:7763. doi: 10.1038/s41467-025-62998-y (PMC12368222; doi:10.1038/s41467-025-62998-y)
Supplement: Supplementary file 1 — Supplementary Information [file 41467_2025_62998_MOESM1_ESM.pdf]

Supplementary information for "In-cell Structure and Variability of Pyrenoid Rubisco"

Nadav Elad[1,2†], Zhen Hou[3†], Maud Dumoux[4], Alireza Ramezani[5], Juan R. Perilla[5], Peijun Zhang[2,3,6*]

[1]Department of Chemical Research Support, Weizmann Institute of Science, Rehovot, Israel.
[2]Diamond Light Source, Harwell Science and Innovation Campus, Didcot, OX11 0DE, UK
[3]Division of Structural Biology, Nuffield Department of Medicine, University of Oxford, Oxford, OX3 7BN, UK
[4] The Rosalind Franklin Institute, Didcot, OX11 0QX, UK
[5]Department of Chemistry and Biochemistry, University of Delaware, Newark, DE 19716, USA
[6]Chinese Academy of Medical Sciences Oxford Institute, University of Oxford, Oxford, OX3 7BN, UK

Detailed methods for real-space refinement of Rubisco coordinates within the cryo-EM map

Rigid-body docking of Rubisco models into cryo-EM density

Initially, rigid-body docking was employed to fit the large subunits (PDB: 1GK8[1]) and small subunits (PDB: 1EJ7[2]) of Rubisco structure into the cryo-EM density. Mutations introduced during crystallization were reverted to the wild-type sequence (UniProt ID P00877 for the large subunit and P00873 for the small subunit) using ChimeraX[3].

Homology modeling of flexible regions of Rubisco

Flexible regions, namely residue 330 to 339 and 462 to 475 of the large subunits, were generated *de novo* using an iterative procedure with Rosetta version 2024.09[4–7]. Pseudo-D4 symmetry (Supplementary Fig. 18) transformation matrices were created using make_symmdef_file.pl binary in Rosetta, with the matrix details provided in Supplementary Table 5. One large subunit and one small subunit were considered as a long chain for the symmetry calculation. First, both loop 6 (T330-R339) and C-terminus (W462-L475) of the large subunit were generated using hybridize mover[8] in Rosetta while the pseudo symmetry was imposed. An elec_dens_fast weight of 35 was provided to Rosetta for calculating the energy score that the model would experience in the presence of the cryo-EM density map. The best structure was selected based on Rosetta energy score and visual inspection; the criteria for visual inspection was how loop 6 (T330-R339) and D473 were fitted to the density. Second, part of the C-terminus (W462-T472) of the large subunit that were out of the cryo-EM density were regenerated once again after restraining the distance between D473 Carbon-Gamma and R134 Carbon-Zeta, and H310 Carbon-Gamma, respectively to guide the model into the cryo-EM density. Flat harmonic potentials[9,10] with a standard deviation of 0.25 and tolerance of 1.0, centered at 4.5 and 5.1 Å, for R134 and H310, respectively, were used to model previously observed interactions between these residues[2]. Next, an iterative local rebuilding procedure was applied using CartesianSampler mover in Rosetta. Backbone segments that appeared incorrect based on agreement with local cryo-EM density and their local strain were detected for rebuilding. A fragment length of 7 was used to control the length of the fragments being resampled. New fragments were accepted for replacement when the RMSD between the endpoints of the fragments was below 1.5 Å.

Molecular Dynamics Flexible Fitting of Rubisco

Starting from the Rosetta-derived model, protonation states of the titratable groups and coordinates of the hydrogen atoms were added using the PDB2PQR[11] at pH 5.8. Counterions were positioned near the protein using the CIONIZE[12] plugin in VMD[13] with the number of ions determined based on the charged residues present in the Rubisco structure. Ions located more than 15 Å from the protein were removed. The system was then solvated with TIP3P[14] water molecules incorporating a 20 Å padding along each principle axis, resulting in a water box measuring 61× 60 × 72 Å. This solvation was performed using solvate plugin in VMD. A salt concentration of 0.150 M KCl was stablished by addition of bulk ions using autoionize plugin in VMD. The protein structure file for the Rubisco, ions, and water molecules were generated with psfgen plugin in VMD. Secondary structure and chiral restraints were generated using cispeptide, ssrestraint, and chirality plugins in VMD. The system was then minimized using a conjugate gradient algorithm with linear search implemented in NAMD2.15alpha2. Subsequently, Molecular dynamics flexible fitting (MDFF)[15,16] simulations were conducted using NAMD_3.0alpha13[17,18] with the CHARMM36 force field[19]. A time step of 2 fs was employed with the r-RESPA integrator, updating nonbonded

interactions every 2 fs, and electrostatic interactions every 4 fs. Long-range electrostatic interactions were computed using the Particle-mesh Ewald (PME) method with a grid spacing equal to 1.0 Å. A cutoff of 12 Å was employed with a switching distance of 10 Å to smoothen the interactions beyond the cutoff. The SHAKE[20] algorithm was applied to restrain all hydrogen bonds. The system temperature was maintained at 300 K using a Langevin thermostat, having a coupling coefficient of 5 ps^{-1}. Random initial velocities were assigned to each atom to generate a Maxwell distribution at temperature 300 K. Pressure control was achieved using Nose-Hoover Langevin barostat, at 1.0135 bar, with decay and period of 100 ps and 200 ps, respectively. The backbone heavy atoms were coupled to the cryo-EM density map through a grid-based biasing-potential[21] with a coupling constant of 0.3 au. A harmonic force were applied on all heavy atoms with a spring constant ramping to 2000 $kcal.mol^{-1}.Å^{-2}$ over 1 ns, to transform and overlap the atomic coordinates. Transformation matrices used during the MDFF are provided in Supplementary Table 6. Secondary structure and chiral restraints were enforced utilizing extraBonds, as implemented in NAMD.

The resulting model was in good agreement with the density as demonstrated by Supplementary Fig. 15a) and supported by the local cross-correlation analysis (Supplementary Fig. 15b-c).

The system obtained from MDFF was then minimized using a conjugate gradient algorithm implemented in NAMD for 40000 steps, ensuring convergence with energy gradient variance falling below 1.0 $kcal.mol^{-1}.Å^{-2}$. During minimization, a coupling constant of 5.0 applied to couple backbone heavy atoms to the cryo-EM density.

Creating a structure fitted at both termini

The MDFF-derived model revealed multiple mappings between atomistic coordinates and the cryo-EM density map at the N- and C-termini of the large subunit. While chains fitted well to the density at the C-terminus (Supplementary Fig. 12b), and others fitted at the N-terminus (Supplementary Fig. 12d), no single chain was fitted at both termini simultaneously. To address the latter, two chains were selected based on visual inspection of their fit to the density at each terminus (Supplementary Figs. 12a-d). Their structures were superimposed by aligning their sequences, then fitting their Carbon-Alpha using the matchmaker command in ChimeraX. Only the residues with secondary structure elements from residue 36 to 139 (beta sheets) were selected for superimposing to obtain better superposition (Supplementary Fig. 12e). The superposition of structures allowed us to combine the coordinates of two chains with minimal deviation of atomic positions (Supplementary Fig. 12f).

As a result, a hybrid large subunit was created by combining the residues from 1 to 103 taken from the chain matching the density at N-terminus, while the rest, residues 104 to 475, were taken from the other chain. The hybrid large subunit made by combining these two chains, was subsequently rigid-body fitted to each large subunit in the MDFF-derived model. Finally, the complete Rubisco structure, built from the hybrid model, was subjected to a further relaxation to remove possible steric clashes using NAMD3.0.1.

Iterative model refinement

The hybrid model underwent further iterative refinement using a unsupervised[22] and supervised protein refinement tool, applying FastRelax[23–25] protocol in Rosetta. The unsupervised refinement tool identifies regions with low molProbity score[26,27] and refined only those regions while keeping the rest of the structure fixed using MoveMap in FastRelax. Similarly, python and Tool Command

Language (TCL) scripts were developed to identify the regions with poor Local-Cross Correlation (LCC) score and apply FastRelax protocol specifically on those regions (Supplementary Fig. 13).

Improving the quality of the beta sheets

Although the protein backbone atoms were well fitted to the density in the refined model, the beta sheets of the model were not predicted properly (Supplementary Fig. 14a-b). In order to enhance the quality of the beta sheets in our model, the transformation matrix from the backbone of beta sheets in a large subunit of PDB 1GK8[1], namely residues 169-171, 199-201, 237-241, 264-268, 290-294, 325-327, 375-379, and 399-40 to the backbone of previously mentioned residues in the refined model were calculated using VMD. Next, these residues were superimposed on the refined model by applying the transformation matrix on them. Then the atomic coordinates of these residues in the refined model were replaced by the coordinates of superimposed residues. Similar procedures were performed on another set of beta sheets, i.e. residues 36-44, 83,89, 97-103, and 130-139.

Second round of Molecular Dynamics Flexible Fitting of Rubisco

The coordinates of the hydrogen atoms were added using the PDB2PQR[11] at pH 5.8. The protons added by PDB2PQR to some of the charged amino acids in the structure, i.e. GLU and ASP, were deleted from the structure. The system was ionized, solvated, and underwent an MDFF simulation following the same procedures described earlier. Alternatively, during the MDFF simulation, a harmonic force with spring constant of 4000 $kcal.mol^{-1}.Å^{-2}$ were applied over 0.5 ns on the heavy atoms of the protein. The MDFF were performed utilizing NAMD3.0.1 with CHARMM36m force field[28]. Lastly the model was minimized with NAMD for 40000 steps. The proteins were not coupled to the cryo-EM density during the final minimization. The final structure indicates good agreement with the cryo-EM density map as illustrated by Fourier Shell Correlation (FSC) plot (Supplementary Fig. 15d) and Local Cross Correlation (LCC) between the structure and the map (Supplementary Fig. 15e-f). The beta sheets in the final model are visualized in (Supplementary Fig. 11c-d).

Supplementary Tables

Supplementary Table 1. Cryo-FIB lamella preparation.

Method	Blind milling	Blind milling
Microscope	Plasma FIB Arctis	Conventional FIB Aquilos 2
Voltage (keV)	30	30
Ion beam source	Argon	Gallium
Sputtering coating prior to milling (seconds)	12	No
GIS coating time (second)	50	30
Bulk milling current	N/A	N/A
Milling current	0.74-2 nA	0.1-0.5 nA
Polishing current	60 pA	30 pA
Sputtering coating post polishing (seconds)	No	No
Fluorescence microscope	iFLM, 100× (TFS)	METEOR, 50× (Delmic)
Number of lamellae prepared	37	29

Supplementary Table 2. Cryo-ET data collection.

Sample	Arctis lamellae	Aquilos 2 lamellae
Microscope	FEI Titan Krios G3	FEI Titan Krios G3
Voltage (keV)	300	300
Detector	Falcon 4i	Falcon 4i
Energy-filter	Selectris X	Selectris X
Slit width (eV)	10	10
Super-resolution mode	No	No
Physical pixel size (Å/pixel)	1.978	1.97
Defocus range (µm)	-2.5 to -4.5, increment 0.3	-2.5 to -4.5, increment 0.3
Acquisition scheme	Dose-Symmetric, -54º/54º, 2º step, group 3	Dose-Symmetric, -54º/54º, 2º step, group 3
Total dose (electrons/$Å^2$)	120	120
Number of frames	6	6
Number of lamellae	37	29
Number of selected tomograms/micrographs	13	13

Supplementary Table 3. Number of subtomograms and resolution of the classes. Class numbers according to the initial classification (Supplementary Fig. 2c) and the number of subtomograms per class are listed in the first and second columns, respectively. The proportion of each class from the total C1-refined dataset is listed in the third column. Classes that did not converge in the C1 refinement were excluded from this dataset and are marked with a minus sign. Resolution of the same classes refined with C1 or D4 symmetries applied (Supplementary Fig. 2d, e), are listed in the fourth and fifth columns, respectively.

**Class**	**Number of Sub-tomograms**	**% of the "good" C1-refined classes**	**C1 resolution at 0.143 FSC cut-off (Å)**	**D4 resolution at 0.143 FSC cut-off (Å)**
1	12,318	8%	24.6	-
2	6,773	5%	23.2	17.1
3	8,317	6%	23.2	-
4	12,275	-	-	-
5	7,165	5%	21.9	-
6	7,797	-	-	-
7	8,339	6%	18.8	12.3
8	15,311	-	-	-
9	9,666	7%	23.2	17.9
10	12,267	8%	19.7	13.6
11	7,841	5%	28.1	-
12	17,713	12%	13.1	8.1
13	7,611	5%	20.7	13.6
14	8,407	6%	21.9	-
15	14,197	-	-	-
16	14,462	10%	20.7	17.1
17	9,587	6%	21.9	14.6
18	17,053	-	-	-
19	11,398	8%	17.1	15.2
20	6,775	5%	22.9	17.1
**Total Sub-tomograms**	215,272	148,639 (100%)	148,639	104,591

Supplementary Table 4. Summary of refinement and validation statistics

	Class 12, D4 map (EMD-52438, PDB 9HVM)
**Data collection and processing**	
Magnification	64,000
Voltage (kV)	300
Electron exposure (e–/Å^2)	120
Defocus range (μm)	-2.5 to -4.5
Pixel size (Å)	1.97
Symmetry imposed	D4
Initial particle images (no.)	515,948 (all classes)
Final particle images (no.)	17,713
Map resolution (Å)<br>FSC threshold	8.1<br>0.143
Map resolution range (Å)	7 to 15.6
**Refinement**	
Initial model used (PDB code)	1GK8, 1EJ7
Model resolution (Å)<br>FSC threshold	8.5<br>0.5
Model resolution range (Å)	8.5 (0.5 cutoff)
Map sharpening B factor (Å^2)	NA
Model composition<br>Non-hydrogen atoms<br>Protein residues<br>Ligands	 37,816<br>4,808<br>0
B factors (Å2)<br>Protein<br>Ligand	
R.m.s. deviations<br>Bond lengths (Å)<br>Bond angles (°)	 0.72<br>1.23
Validation<br>MolProbity score<br>Clashscore<br>Poor rotamers (%)	 1.52<br>0<br>4
Ramachandran plot<br>Favored (%)<br>Allowed (%)<br>Disallowed (%)	 90<br>7<br>3

Supplementary Table 5. Rotation matrix used by Rosetta during rebuilding loop 6 and C-terminus.

$$\begin{bmatrix} 1.00 & 0.00 & 0.00 \\ 0.00 & 1.00 & 0.00 \\ 282.20 & 241.06 & 253.39 \end{bmatrix} \begin{bmatrix} -1.00 & 0.01 & -0.01 \\ -0.01 & -1.00 & 0.03 \\ 221.83 & 263.72 & 252.94 \end{bmatrix}$$

$$\begin{bmatrix} -1.00 & -0.02 & 0.00 \\ -0.02 & 1.00 & 0.00 \\ 221.91 & 240.77 & 251.14 \end{bmatrix} \begin{bmatrix} 1.00 & 0.01 & 0.01 \\ 0.01 & -1.00 & 0.02 \\ 282.15 & 263.32 & 251.50 \end{bmatrix}$$

$$\begin{bmatrix} 0.00 & -1.00 & 0.00 \\ 1.00 & 0.00 & 0.00 \\ 241.13 & 221.88 & 253.10 \end{bmatrix} \begin{bmatrix} 0.01 & 1.00 & -0.03 \\ -1.00 & 0.01 & -0.01 \\ 263.11 & 282.45 & 252.72 \end{bmatrix}$$

$$\begin{bmatrix} 0.01 & -1.00 & 0.00 \\ -1.00 & -0.01 & 0.00 \\ 263.34 & 221.79 & 250.79 \end{bmatrix} \begin{bmatrix} 0.00 & 1.00 & -0.03 \\ 1.00 & 0.00 & 0.01 \\ 240.93 & 282.72 & 250.11 \end{bmatrix}$$

Supplementary Table 6. Transformation matrices used for imposing symmetry during MDFF simulation.

$$\begin{bmatrix} 1.00 & 0.00 & 0.00 & 0.00 \\ 0.00 & 1.00 & 0.00 & 0.00 \\ 0.00 & 0.00 & 1.00 & 0.00 \\ 0.00 & 0.00 & 0.00 & 1.00 \end{bmatrix} \quad \begin{bmatrix} -1.00 & 0.00 & 0.00 & 503.67 \\ 0.00 & -1.00 & 0.00 & 504.83 \\ 0.00 & 0.00 & 1.00 & 0.09 \\ 0.00 & 0.00 & 0.00 & 1.00 \end{bmatrix}$$

$$\begin{bmatrix} -1.00 & 0.01 & -0.01 & 505.01 \\ 0.01 & 1.00 & -0.01 & 1.45 \\ 0.01 & -0.01 & -1.00 & 505.10 \\ 0.00 & 0.00 & 0.00 & 1.00 \end{bmatrix} \quad \begin{bmatrix} 1.00 & 0.00 & -0.01 & 3.24 \\ 0.00 & -1.00 & 0.00 & 506.92 \\ -0.01 & 0.00 & -1.00 & 505.45 \\ 0.00 & 0.00 & 0.00 & 1.00 \end{bmatrix}$$

$$\begin{bmatrix} 0.00 & -1.00 & 0.01 & 502.61 \\ 1.00 & 0.00 & 0.00 & 0.14 \\ 0.00 & 0.01 & 1.00 & -1.37 \\ 0.00 & 0.00 & 0.00 & 1.00 \end{bmatrix} \quad \begin{bmatrix} -0.01 & 1.00 & 0.00 & 0.88 \\ -1.00 & -0.01 & 0.01 & 505.14 \\ 0.01 & 0.00 & 1.00 & -0.21 \\ 0.00 & 0.00 & 0.00 & 1.00 \end{bmatrix}$$

$$\begin{bmatrix} 0.00 & -1.00 & 0.00 & 504.16 \\ -1.00 & 0.00 & 0.00 & 505.20 \\ 0.00 & 0.00 & -1.00 & 503.33 \\ 0.00 & 0.00 & 0.00 & 1.00 \end{bmatrix} \quad \begin{bmatrix} 0.01 & 1.00 & -0.01 & -1.07 \\ 1.00 & -0.01 & 0.00 & 3.48 \\ 0.00 & -0.01 & -1.00 & 506.68 \\ 0.00 & 0.00 & 0.00 & 1.00 \end{bmatrix}$$

<u>**Supplementary Figures**</u>

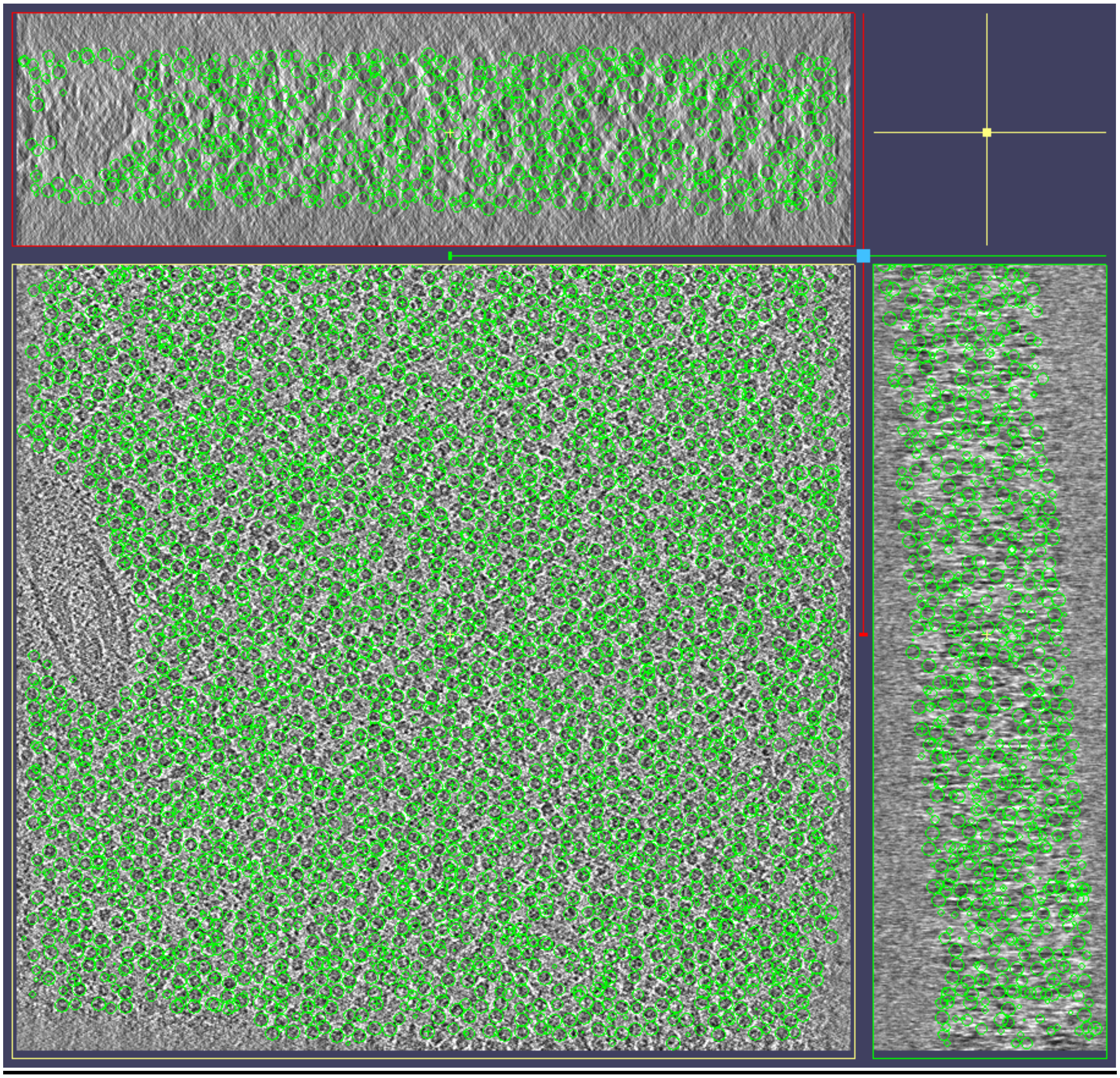

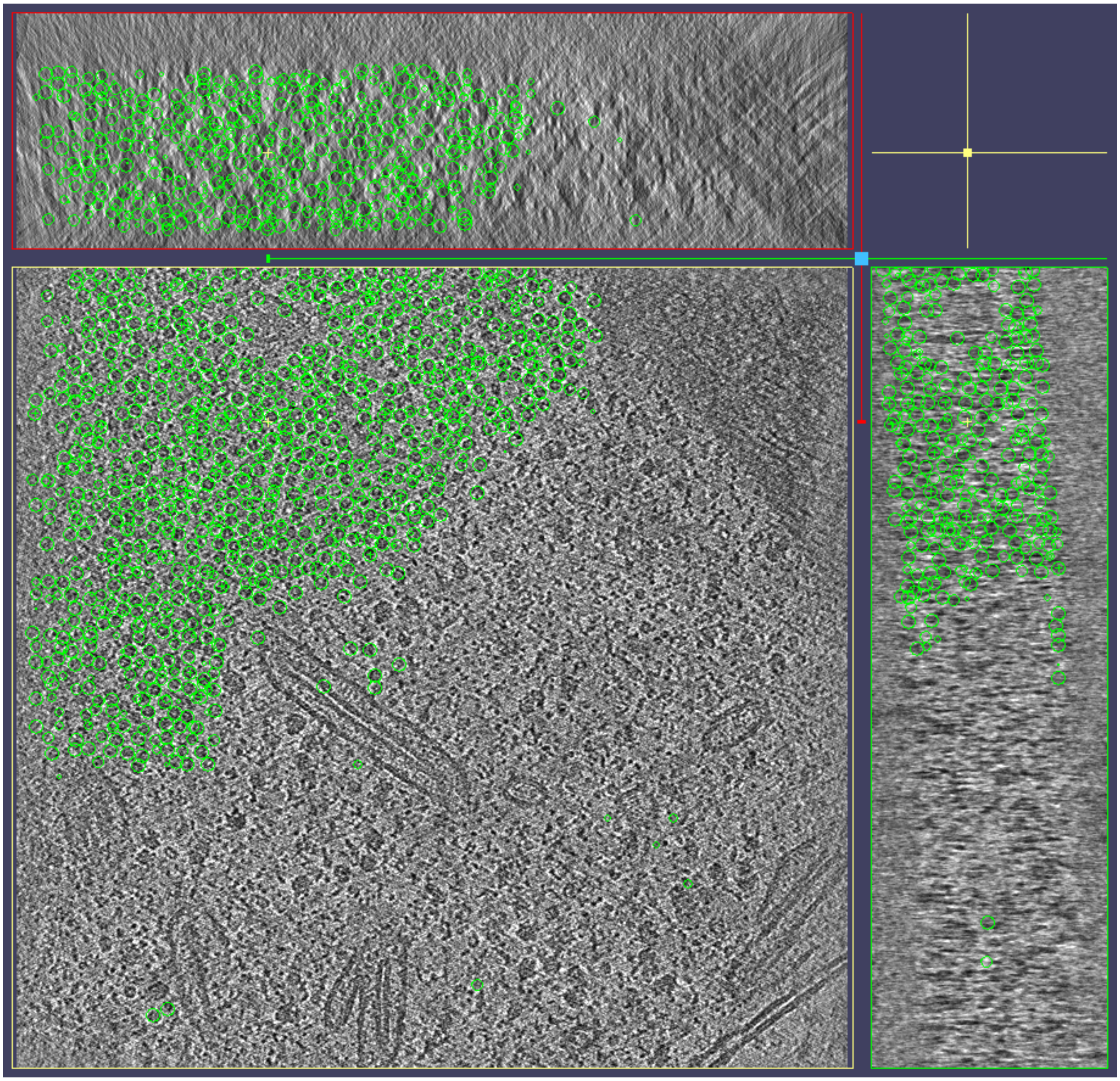

Supplementary Fig. 1. Rubico particle picking from tomograms using deep-learning based crYOLO software[29]. Shown are XY, XZ and YZ slices from two different tomograms ("XYZ view" in IMOD's program 3dmod[30]). The centers of the green spheres mark the Rubisco coordinates, as identified by crYOLO prediction. The spheres' diameter is 8.3 nm, and the slices' thickness is 3.5 nm.

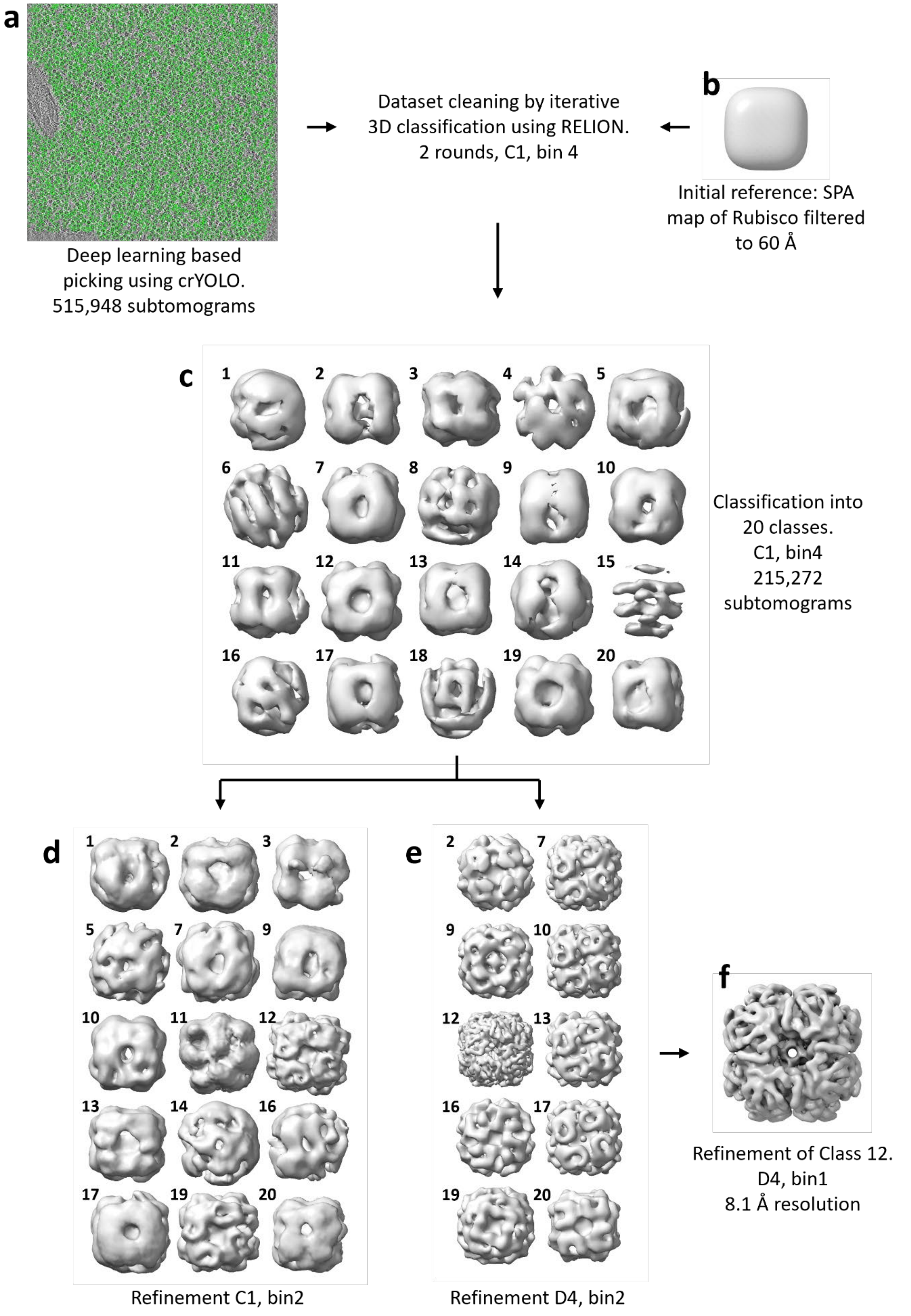
a
Deep learning based
picking using crYOLO.
515,948 subtomograms
Dataset cleaning by iterative
3D classification using RELION.
2 rounds, C1, bin 4
b
Initial reference: SPA
map of Rubisco filtered
to 60 Å
c
1
2
3
4
5
6
7
8
9
10
11
12
13
14
15
16
17
18
19
20
Classification into
20 classes.
C1, bin4
215,272
subtomograms
d
1
2
3
5
7
9
10
11
12
13
14
16
17
19
20
Refinement C1, bin2
e
2
7
9
10
12
13
16
17
19
20
Refinement D4, bin2
f
Refinement of Class 12.
D4, bin1
8.1 Å resolution

Supplementary Fig. 2. STA processing pipeline and the main maps produced. a, Particle picking from tomograms. **b,** Initial reference used for iterative 3D classification in RELION. **c,** Classification following two rounds of cleaning “bad” particles. All twenty classes are shown. **d and e,** Refinement with C1 (d) and D4 (e) symmetries applied. Refinements that converged and feature overall Rubisco structure are shown. Map numbers correspond to the 20 classes in panel (c) above. The number of subtomograms in each class and maps’ resolution are summarized in Supplementary Table 3. **f,** The best-resolved map, refined from class 12.

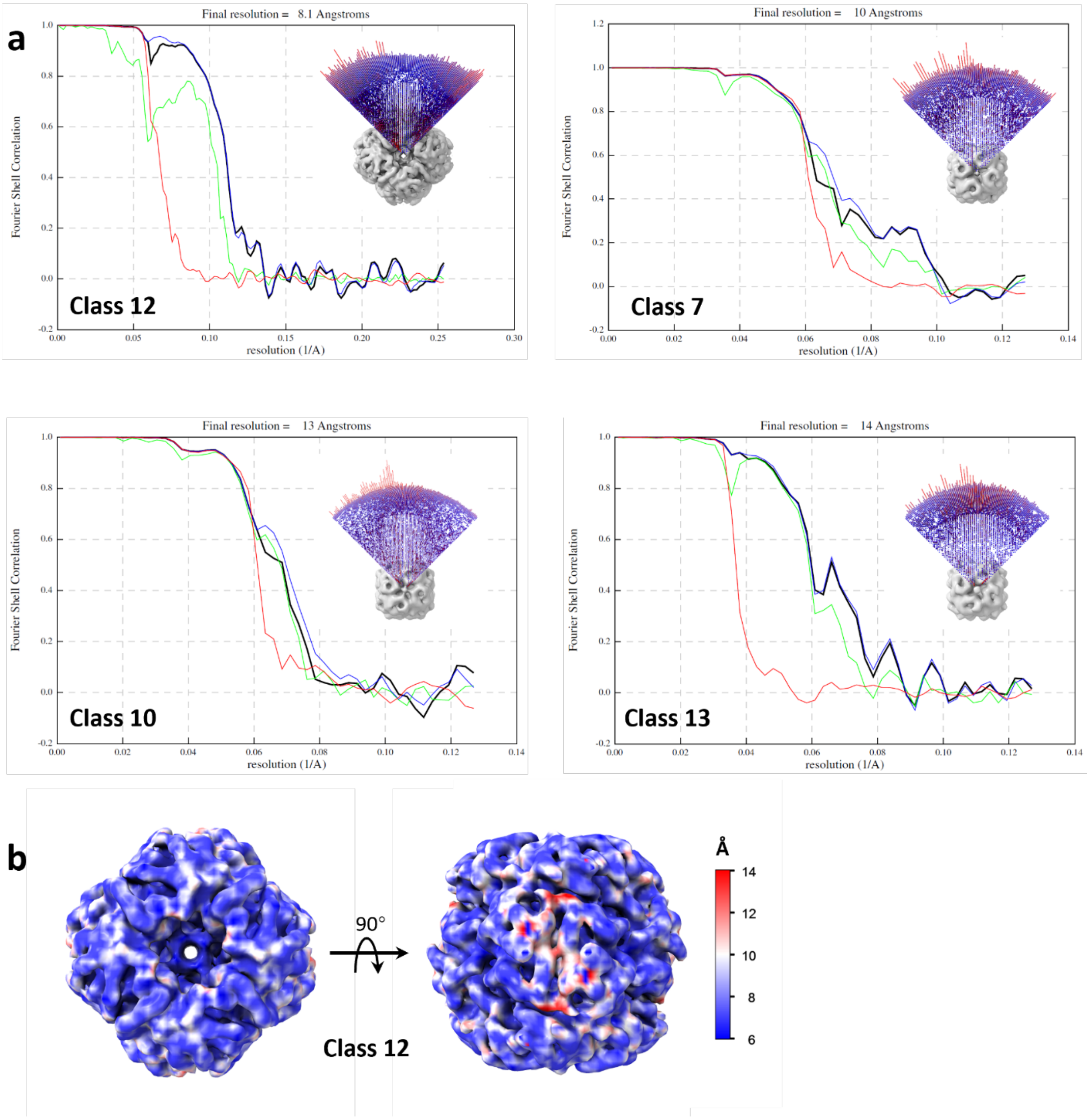

Supplementary Fig. 3. Fourier Shell Correlation (FSC) and angular distribution plots for the best-resolved D4 symmetric 3D classes. **a**, Curves include corrected FSC (black), unmasked maps FSC (green), masked maps FSC (blue) and phase randomized (red). **b**, Class 12 map colored according to local resolution estimate.

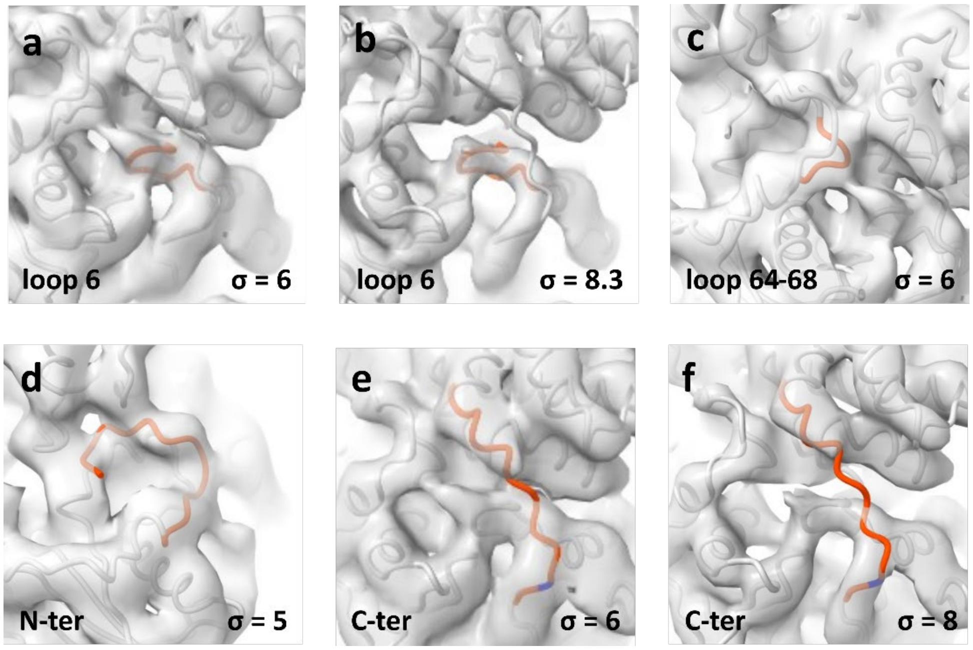

Supplementary Fig. 4. Class 12 map density at key active site fragments. The relevant fragments are colored red and indicated in each panel, along with the map contour level (a-d). Asp473 within the C-terminus is colored blue (e, f).

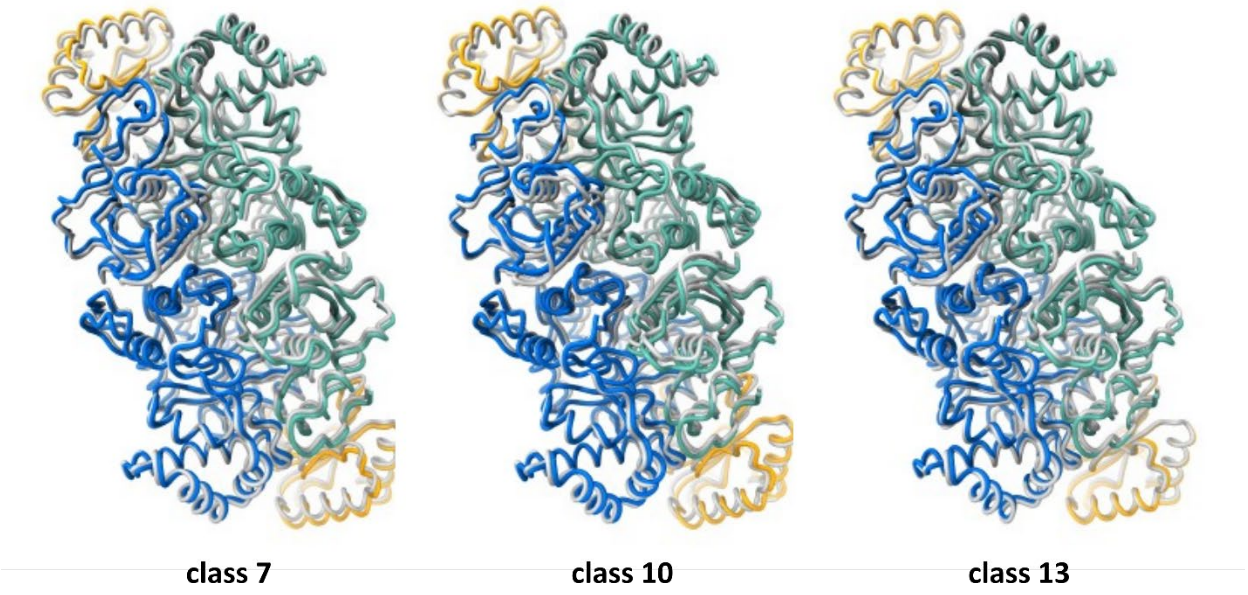

Supplementary Fig. 5. Whole domain movements in classes. The class 12 MDFF model was fitted into the maps of classes 7, 10 and 13. These maps are of lower resolutions, therefore whole domains were fitted as rigid bodies. Domains include the large subunits N-termini (aa 7-148), large subunits C-termini (aa 149-477) and the small subunits. Shown are two large subunits (blue and green) and two small subunits (orange) from each fitted model. The original class 12 MDFF model (grey) is overlayed for reference.

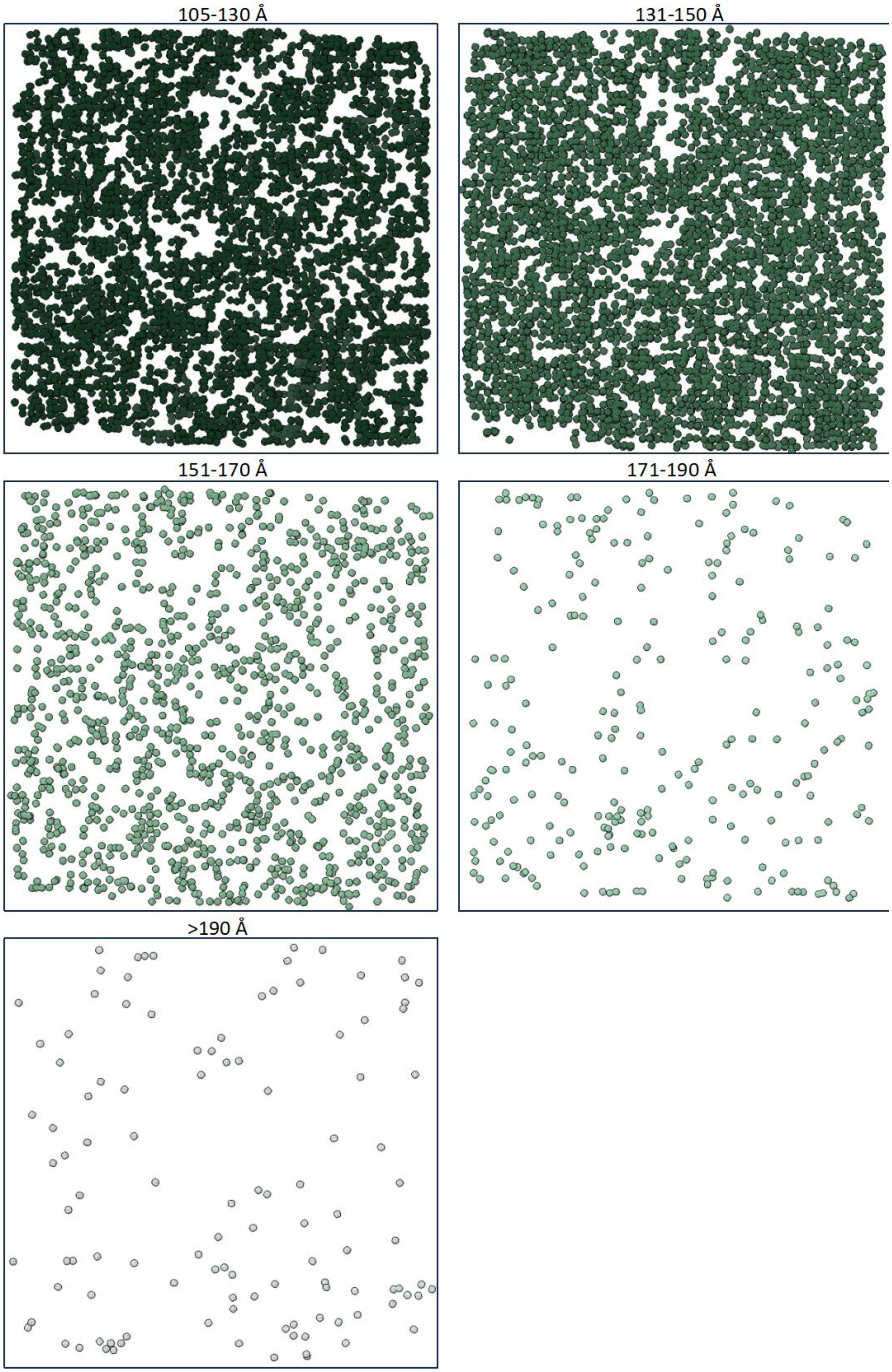

Supplementary Fig. 6. Individual mapping-back of Rubisco of different pair-wise distances in the pyrenoid of the tomogram in Figure 1b.

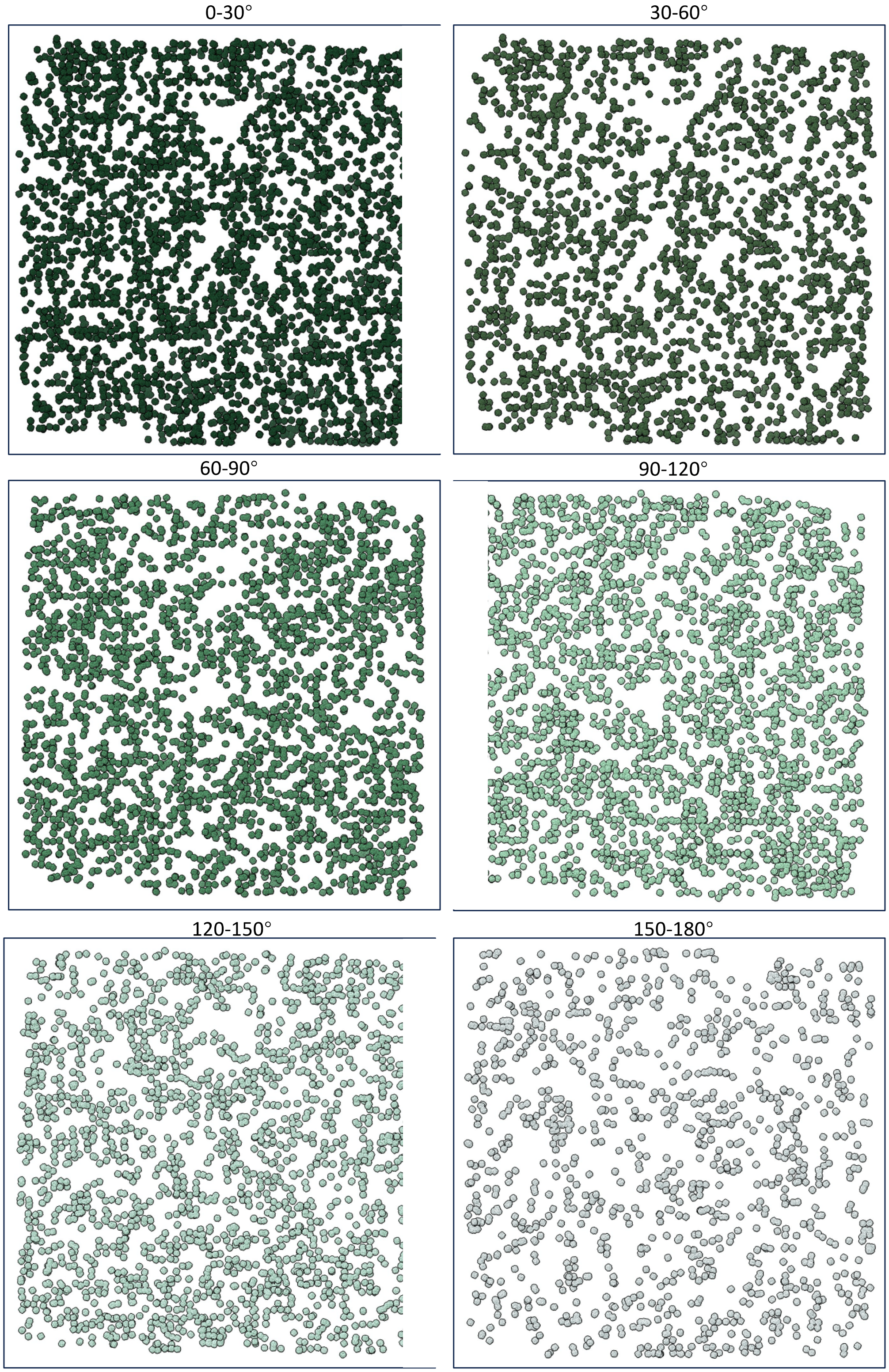

Supplementary Fig. 7. Individual mapping-back of Rubisco of different paired-wise angles in the pyrenoid of the tomogram in Figure 1b.

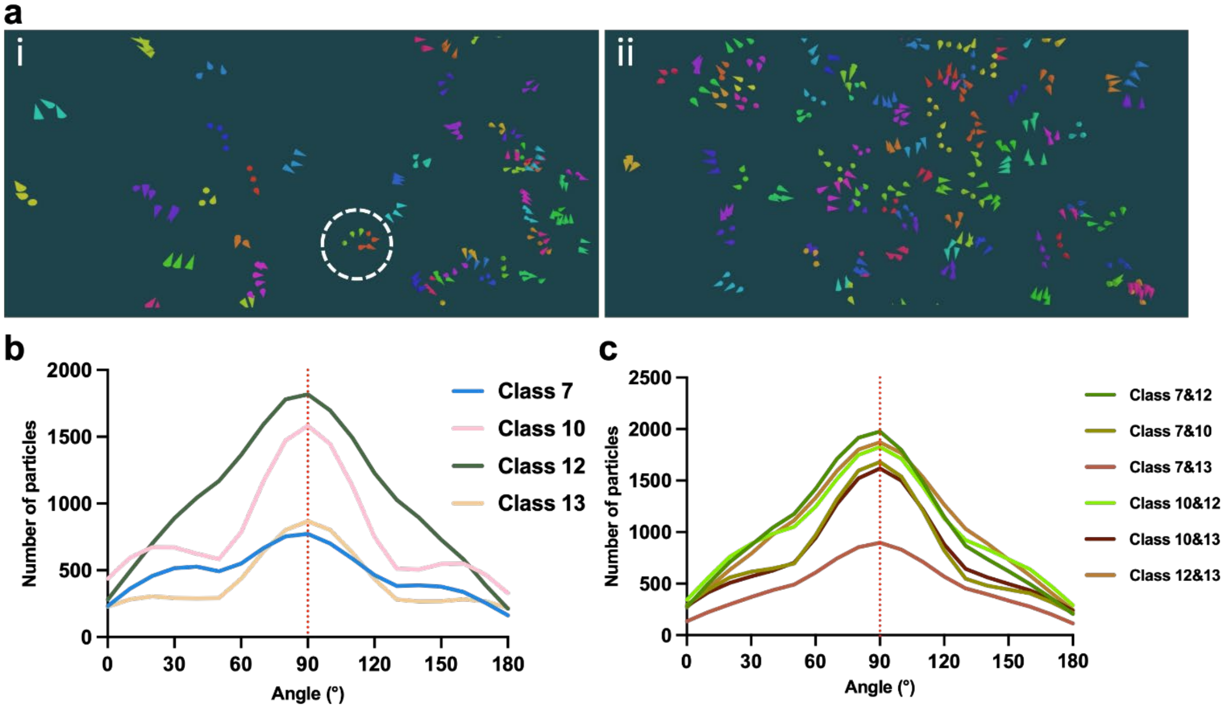

Supplementary Fig. 8. Analysis of Rubisco distribution based on clusters. a, Two representative mapping-back plots of Rubisco particles in two tomograms visualized in MagpiEM. The pointy end indicates the Rubisco orientation, and different clusters are indicated by different colours accordingly. The spiral arrangement of Rubiscos is highlighted in the white dashed circle in (i). The thresholding parameter for clusters is the same as described in **Figure 5e**. **b**, Distribution of pair-wise angles of the nearest neighbours in the best-resolved four classes. **c**, Distribution of pair-wise angles of the nearest neighbours across the best-resolved four classes. The distribution peaks are indicated by dashed lines (*n* of class 7 = 8,339, *n* of class 10 = 12,267, *n* of class 12 = 17,713, *n* of class 13 = 7,611, *n* of tomograms = 26).

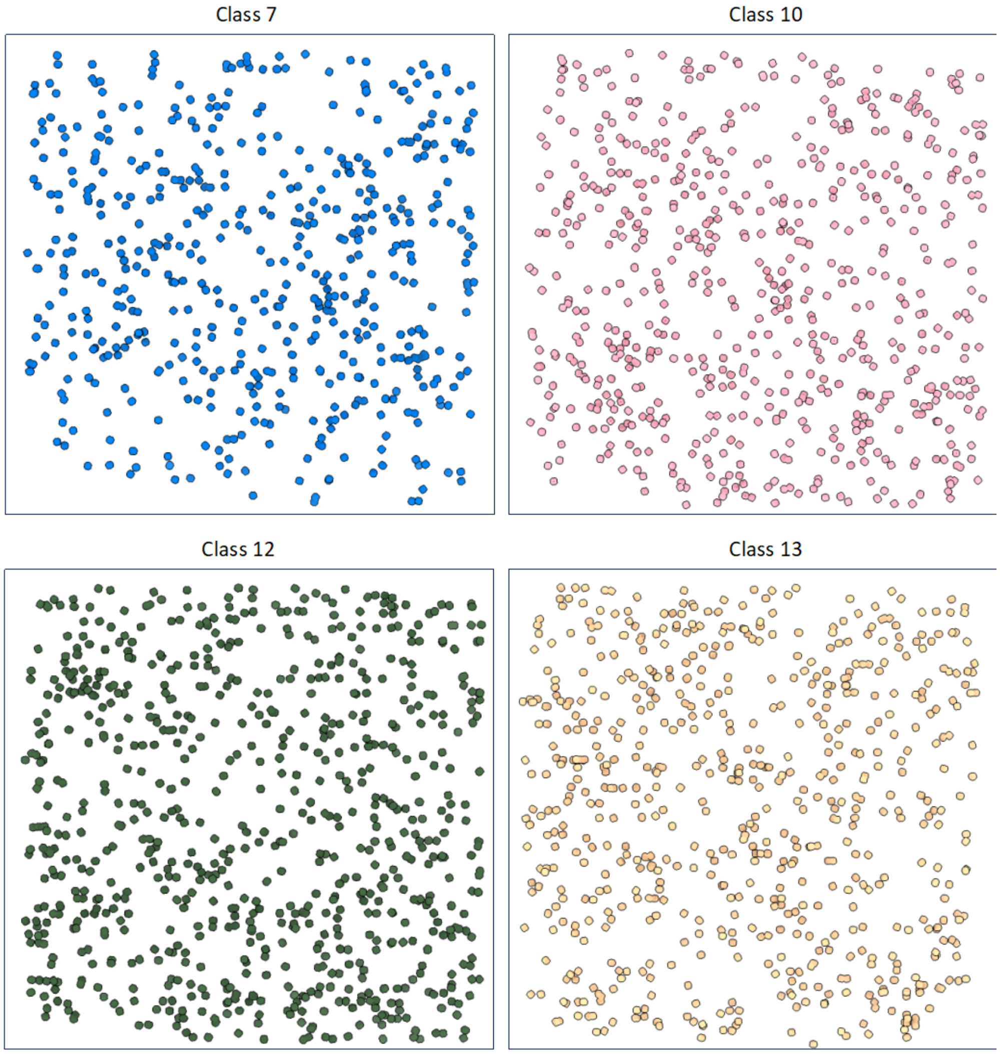

Supplementary Fig. 9. Individual Mapping-back of Rubisco of different classes in the pyrenoid of the tomogram in Figure 1b.

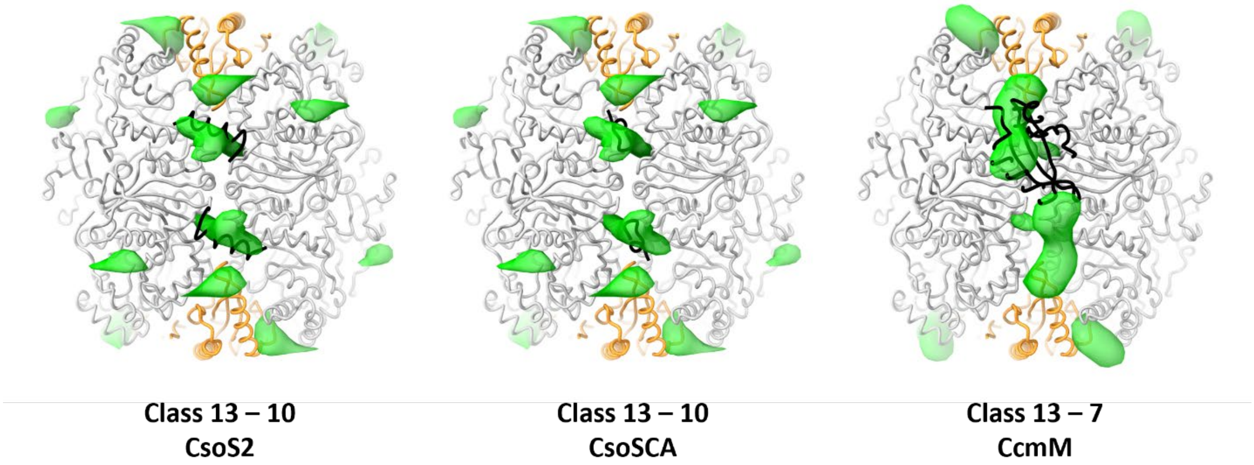

Supplementary Fig. 10. Overlay of difference maps and published Rubisco binding proteins at the large subunit dimer interface. Difference maps (green) are the same as in **Figure 3**. Binding proteins (black) include CsoS2 (PDB 6UEW[31]) and CsoSCA (PDB 7SMK[32]) from α-carboxysomes, and CcmM (PDB 6HBC[33]) from β-carboxysomes. The latter binds asymmetrically to the dimer interface, whereas the difference map originates from a D4-symmetrized map. Positioning of the binding proteins was done by overlaying their corresponding Rubisco coordinates in the structures onto the class 12 MDFF model (colored gray and orange).

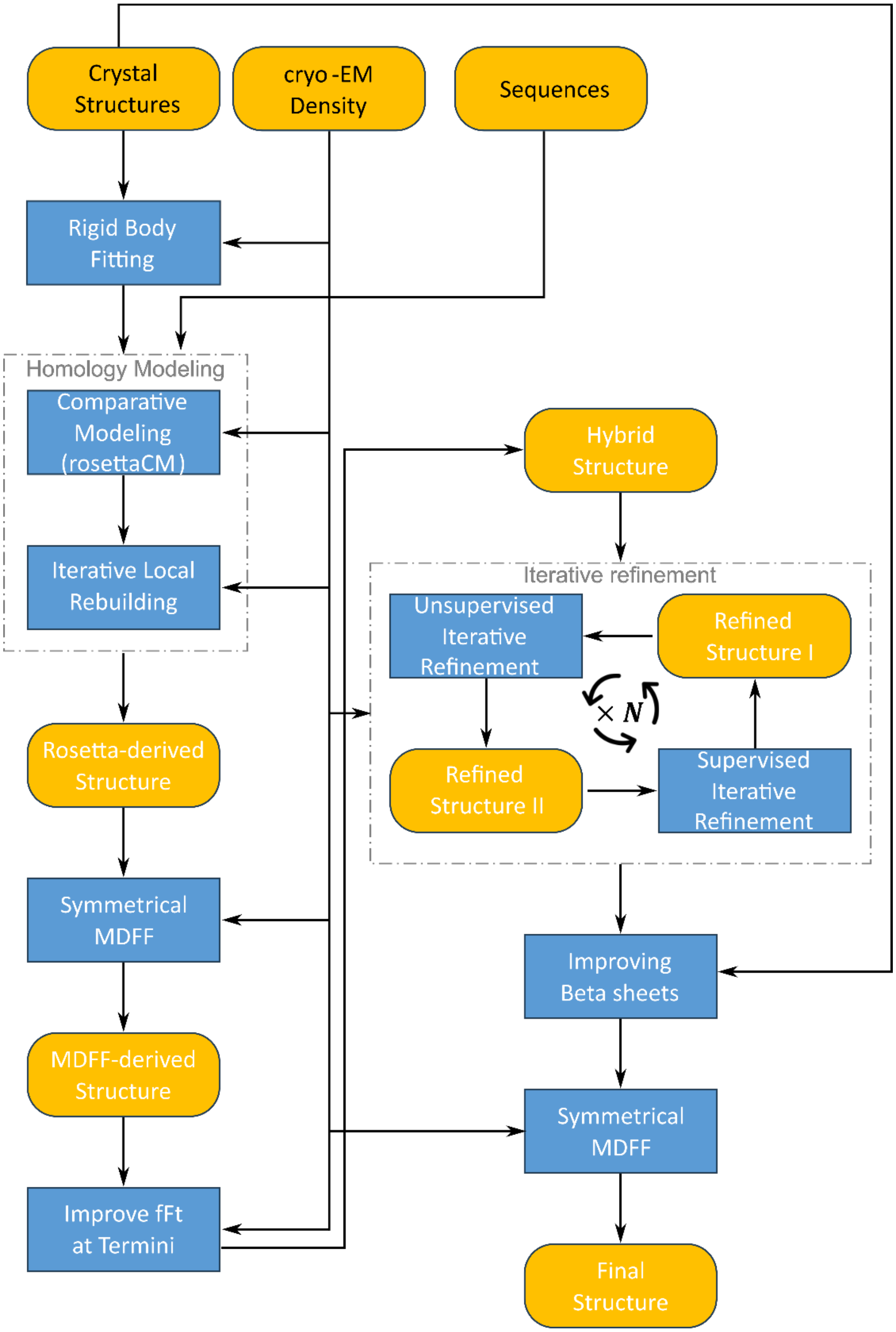

Supplementary Fig. 11. Flowchart for coordinates refinement, integrative modeling and molecular dynamics flexible fitting simulations.

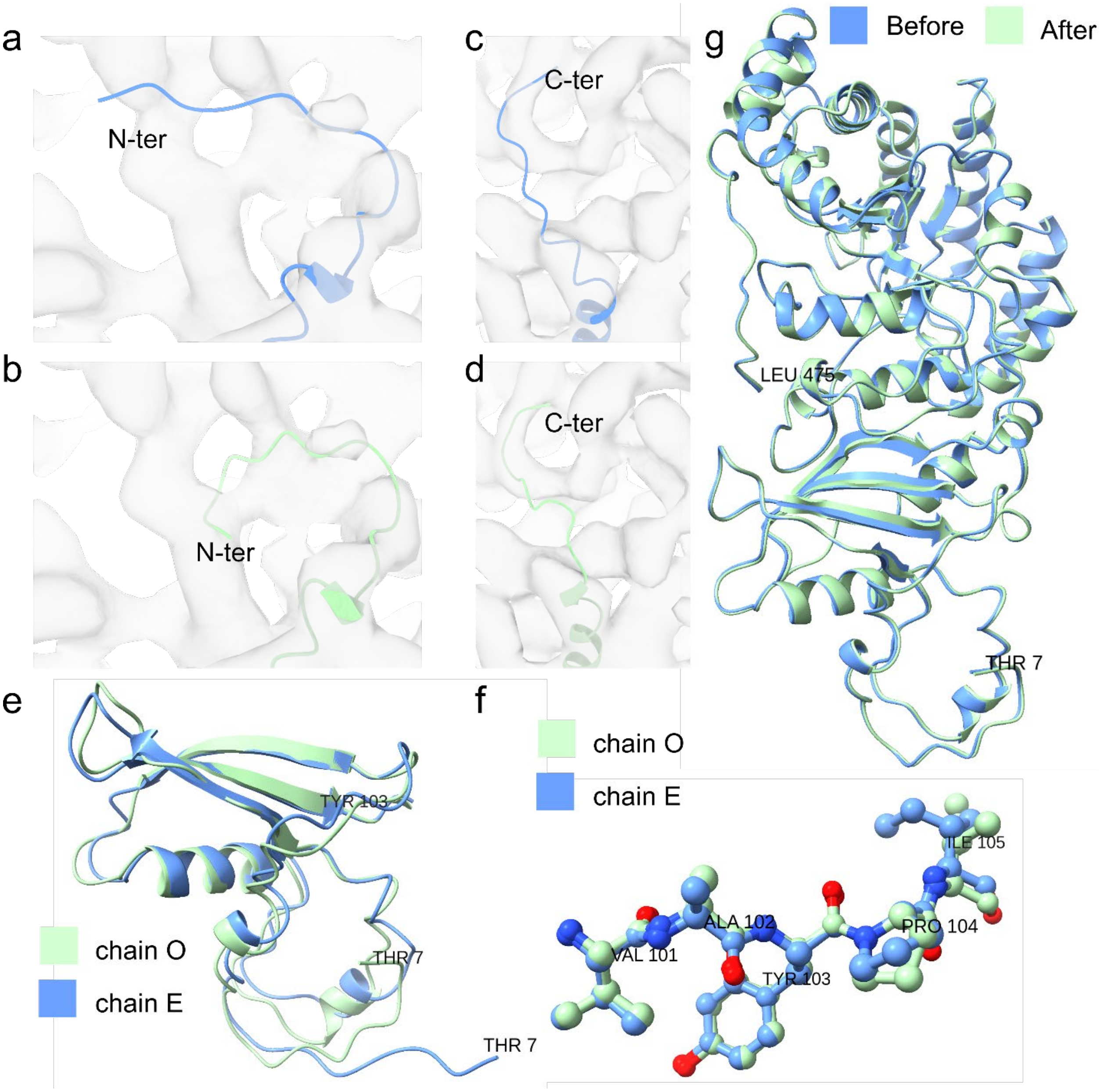

Supplementary Fig. 12. Creating a hybrid model fitted to the density at both termini. Chain O fitted well to the density at the N-terminus, whereas chain E fitted well at the C-terminus. Therefore, a hybrid was created from the well-fitted termini before the refinement of the final model, as described in the Supplementary Information methods. **a, c** Visualization of (a) N-terminus and (c) C-terminus of chain E (blue). **b, d** Visualization of (b) N-terminus and (d) C-terminus of chain O (green). **e** Superposition of both chains, visualized by secondary structure from residue 7 to 145. **f** Ball and stick visualization of the superposition around residue 103. **g** Superposition of the hybrid large subunit before (blue) and after (green) minimization.

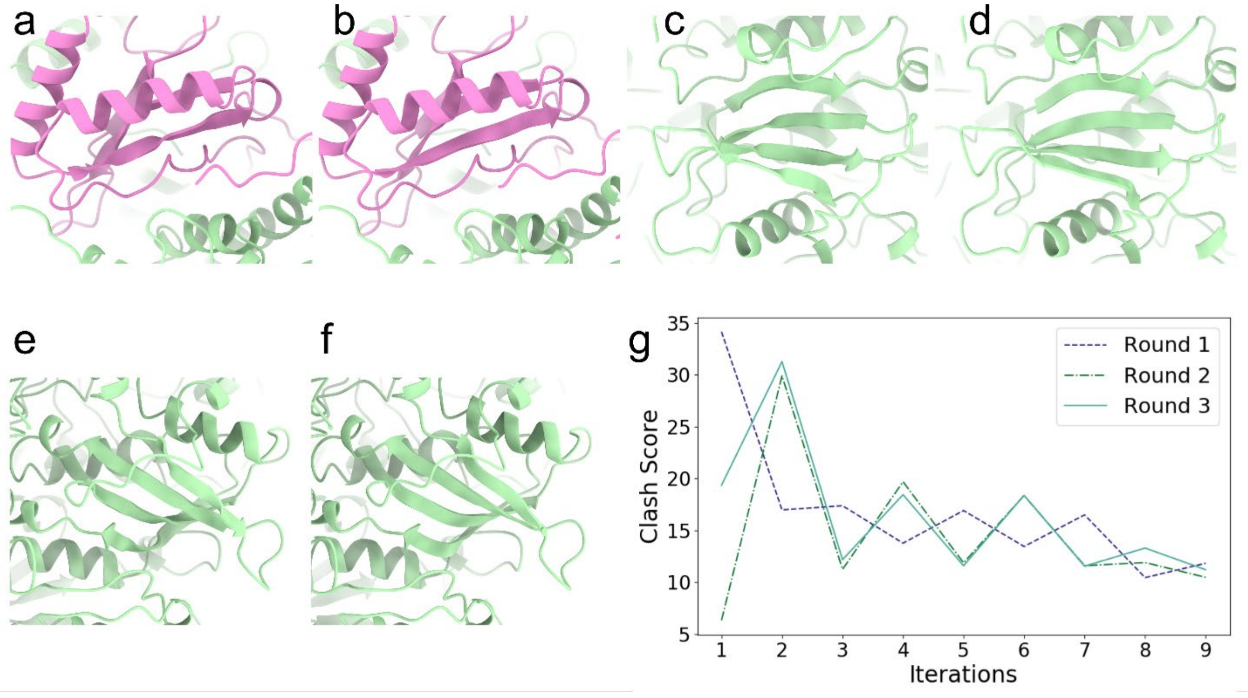

Supplementary Fig. 13. Model improvement by iterative refinements. Instances of regions represented by the secondary structure where the refinement tool improved the model, (a, c, e) before and (b, d, f) after applying the refinement tool. **g** Clash score of the structure for iterations of refinement tool.

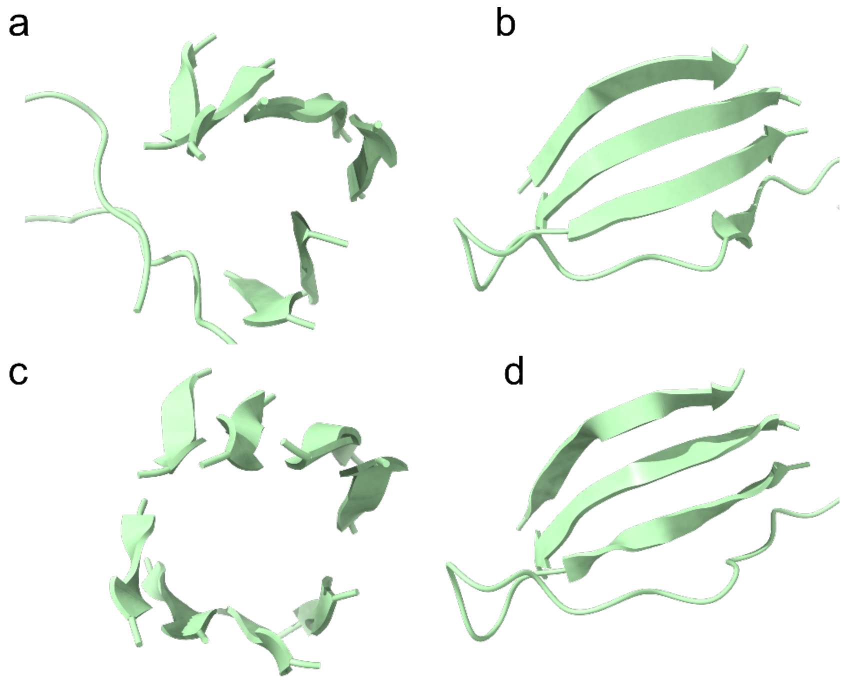

Supplementary Fig. 14. Improving secondary structures. Beta sheets in the large subunit in refined model (a, b) and final model (c, d)

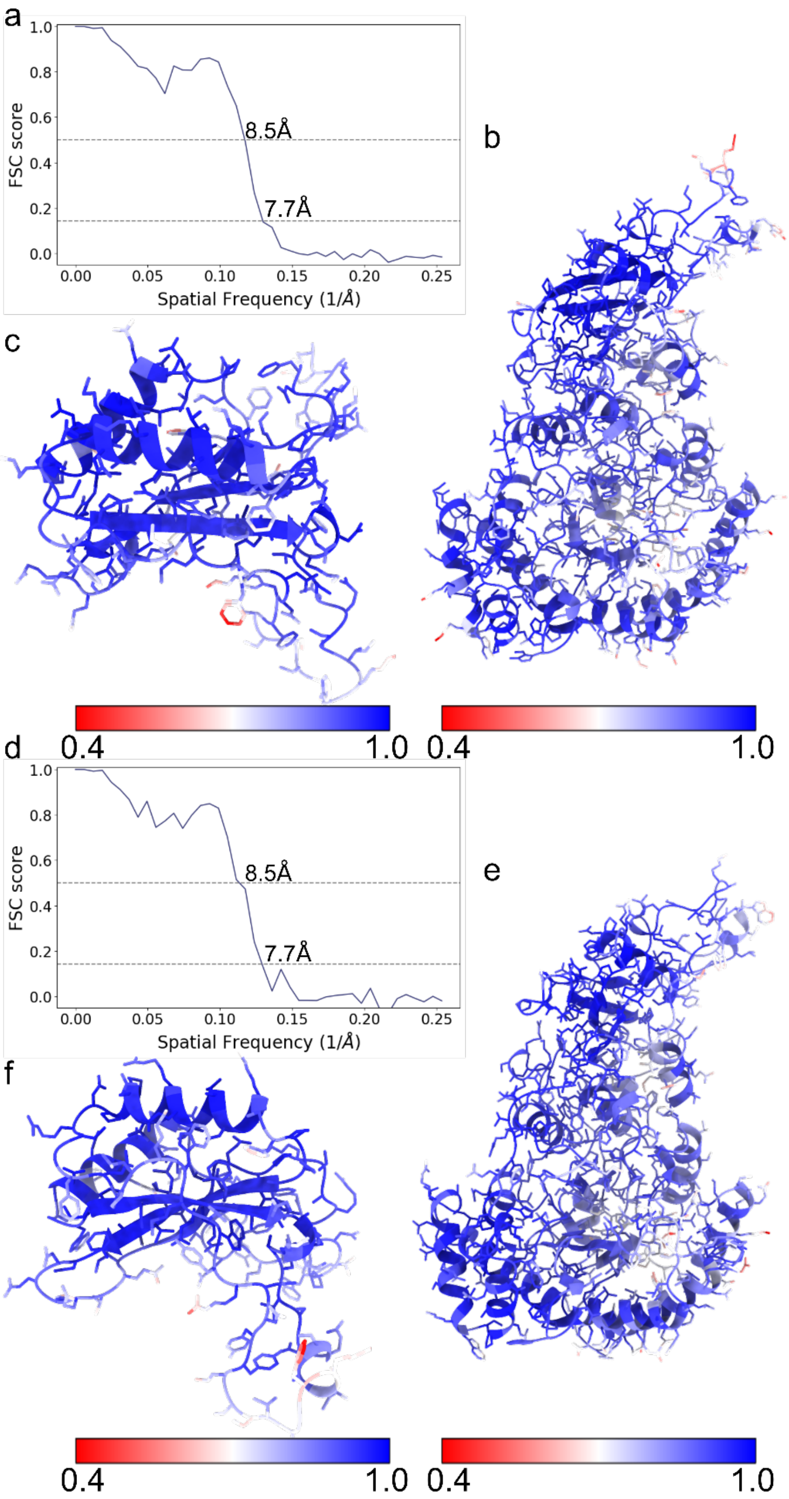

Supplementary Fig. 15. Quantitative assessment of model agreement to the experimental data. a, d FSC plot for Rubisco model to density (a) after MDFF simulation and (d) the final structure. **b, c, e, f** Local-Cross correlation (LCC) of the Rubisco structure and cryo-EM density map after MDFF simulation for (b) large and (c) small subunits, and the final structure for (e) large and small subunits (f).

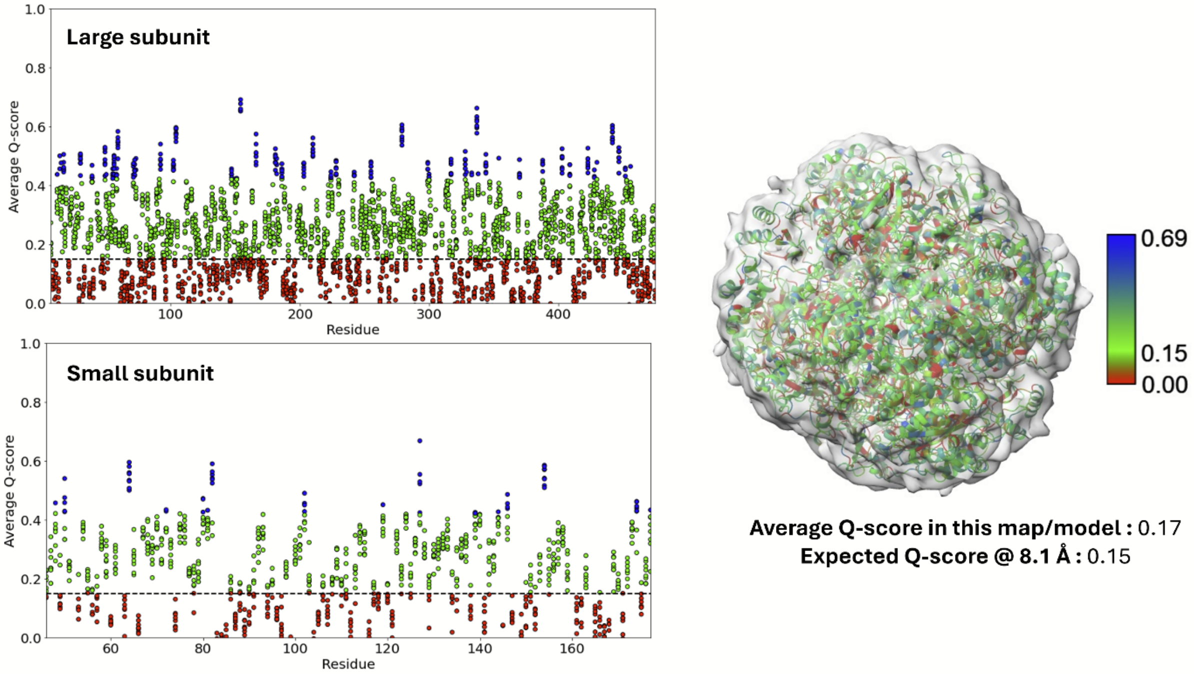

Supplementary Fig. 16. Q-Score analysis per residue. While the Q score varies with the fitting of each residue, the average Q score (0.17, dotted line) for the map/model pair is above the expected for 8.1A resolution (0.15), which means overall the model has good fitting to the density.

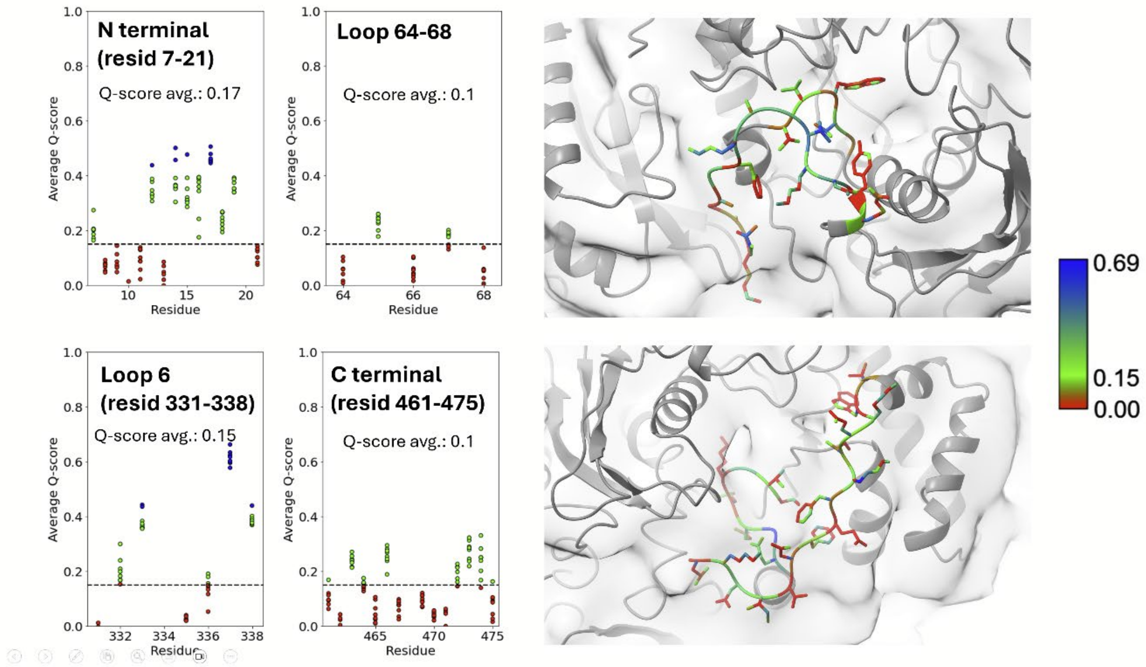

Supplementary Fig. 17. Q-Score analysis on the active site of Rubisco. The Q-scores are within acceptable ranges for the given density for the N terminal and loop 6. Lower Q scores are measured for the Loop 64-68 and the C-terminal region. In particular, Glu468 in the C-terminal region has a negative Q-score (which indicates map values increases away from the position of the residue). Q-scores are known to be lower with low-resolution maps and with flexible residues indicating the flexible nature of Glu468. The dotted line marks the average Q score for the whole model (0.17).

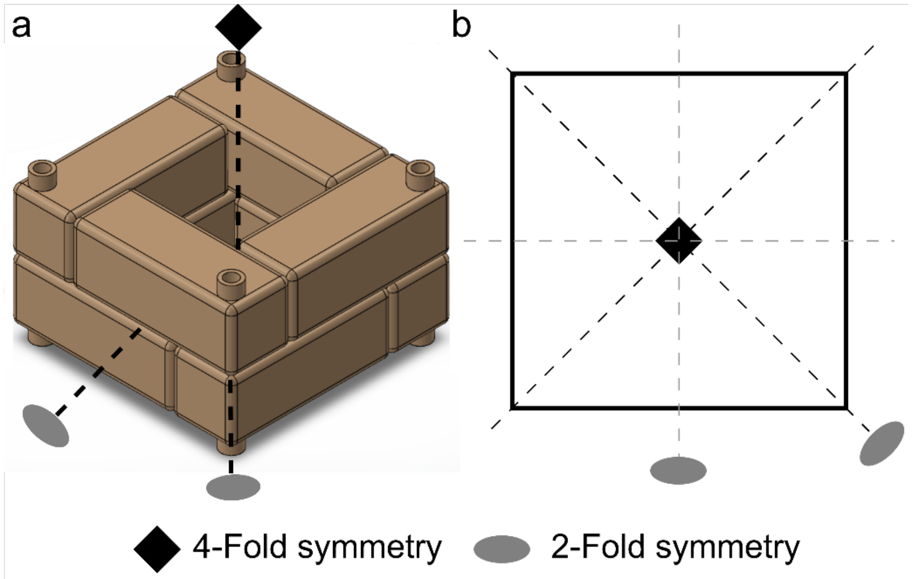

Supplementary Fig. 18. D4 symmetry: eight subunits related to each other by one 4-fold axis and two 2-fold axes.

References

1. Taylor, T. C., Backlund, A., Bjorhall, K., Spreitzer, R. J. & Andersson, I. First Crystal Structure of Rubisco from a Green Alga,*Chlamydomonas reinhardtii* *. *Journal of Biological Chemistry* **276**, 48159–48164 (2001).
2. Duff, A. P., Andrews, T. J. & Curmi, P. M. G. The transition between the open and closed states of rubisco is triggered by the inter-phosphate distance of the bound bisphosphate1. *Journal of Molecular Biology* **298**, 903–916 (2000).
3. Goddard, T. D. *et al.* UCSF ChimeraX: Meeting modern challenges in visualization and analysis. *Protein science* **27**, 14–25 (2018).
4. Loquet, A. *et al.* Atomic model of the type III secretion system needle. *Nature* **486**, 276–279 (2012).
5. Rohl, C. A., Strauss, C. E. M., Misura, K. M. S. & Baker, D. Protein Structure Prediction Using Rosetta. in *Methods in Enzymology* vol. 383 66–93 (Academic Press, 2004).
6. Fleishman, S. J. *et al.* RosettaScripts: A Scripting Language Interface to the Rosetta Macromolecular Modeling Suite. *PLOS ONE* **6**, e20161 (2011).
7. Demers, J.-P. *et al.* High-resolution structure of the Shigella type-III secretion needle by solid-state NMR and cryo-electron microscopy. *Nat Commun* **5**, 4976 (2014).
8. Song, Y. *et al.* High-resolution comparative modeling with RosettaCM. *Structure* **21**, 1735–1742 (2013).
9. Jeliazkov, J. R., Frick, R., Zhou, J. & Gray, J. J. Robustification of RosettaAntibody and Rosetta SnugDock. *PLOS ONE* **16**, e0234282 (2021).
10. King, C. A. & Bradley, P. Structure-based prediction of protein–peptide specificity in rosetta. *Proteins: Structure, Function, and Bioinformatics* **78**, 3437–3449 (2010).
11. Dolinsky, T. J., Nielsen, J. E., McCammon, J. A. & Baker, N. A. PDB2PQR: an automated pipeline for the setup of Poisson–Boltzmann electrostatics calculations. *Nucleic Acids Research* **32**, W665–W667 (2004).
12. Stone, J. E. *et al.* Accelerating molecular modeling applications with graphics processors. *Journal of Computational Chemistry* **28**, 2618–2640 (2007).
13. Humphrey, W., Dalke, A. & Schulten, K. VMD: Visual molecular dynamics. *Journal of Molecular Graphics* **14**, 33–38 (1996).
14. Jorgensen, W. L., Chandrasekhar, J., Madura, J. D., Impey, R. W. & Klein, M. L. Comparison of simple potential functions for simulating liquid water. *The Journal of Chemical Physics* **79**, 926–935 (1983).
15. Goh, B. C. *et al.* Computational Methodologies for Real-Space Structural Refinement of Large Macromolecular Complexes. *Annual Review of Biophysics* **45**, 253–278 (2016).
16. Trabuco, L. G., Villa, E., Mitra, K., Frank, J. & Schulten, K. Flexible Fitting of Atomic Structures into Electron Microscopy Maps Using Molecular Dynamics. *Structure* **16**, 673–683 (2008).
17. Phillips, J. C. *et al.* Scalable molecular dynamics on CPU and GPU architectures with NAMD. *The Journal of Chemical Physics* **153**, 044130 (2020).
18. Phillips, J. C. *et al.* Scalable molecular dynamics with NAMD. *Journal of Computational Chemistry* **26**, 1781–1802 (2005).
19. Best, R. B. *et al.* Optimization of the additive CHARMM all-atom protein force field targeting improved sampling of the backbone φ, ψ and side-chain $\chi(1)$ and $\chi(2)$ dihedral angles. *J Chem Theory Comput* **8**, 3257–3273 (2012).

20. Kräutler, V., van Gunsteren, W. F. & Hünenberger, P. H. A fast SHAKE algorithm to solve distance constraint equations for small molecules in molecular dynamics simulations. *Journal of Computational Chemistry* **22**, 501–508 (2001).
21. Wells, D. B., Abramkina, V. & Aksimentiev, A. Exploring transmembrane transport through α-hemolysin with grid-steered molecular dynamics. *The Journal of Chemical Physics* **127**, 125101 (2007).
22. Bryer, A. J., Rey, J. S., Xu, C., Hadden-Perilla, J. A. & Perilla, J. R. Unsupervised Refinement of Protein Structures. (2023) doi:10.1039/BK9781837670154-00327.
23. Tyka, M. D. *et al.* Alternate States of Proteins Revealed by Detailed Energy Landscape Mapping. *Journal of Molecular Biology* **405**, 607–618 (2011).
24. Khatib, F. *et al.* Algorithm discovery by protein folding game players. *Proceedings of the National Academy of Sciences* **108**, 18949–18953 (2011).
25. Maguire, J. B. *et al.* Perturbing the energy landscape for improved packing during computational protein design. *Proteins: Structure, Function, and Bioinformatics* **89**, 436–449 (2021).
26. Davis, I. W., Murray, L. W., Richardson, J. S. & Richardson, D. C. MOLPROBITY: structure validation and all-atom contact analysis for nucleic acids and their complexes. *Nucleic Acids Res* **32**, W615-9 (2004).
27. Davis, I. W. *et al.* MolProbity: all-atom contacts and structure validation for proteins and nucleic acids. *Nucleic Acids Research* **35**, W375–W383 (2007).
28. Huang, J. *et al.* CHARMM36m: an improved force field for folded and intrinsically disordered proteins. *Nature Methods* **14**, 71–73 (2017).
29. Wagner, T. *et al.* SPHIRE-crYOLO is a fast and accurate fully automated particle picker for cryo-EM. *Commun Biol* **2**, 1–13 (2019).
30. Kremer, J. R., Mastronarde, D. N. & McIntosh, J. R. Computer Visualization of Three-Dimensional Image Data Using IMOD. *Journal of Structural Biology* **116**, 71–76 (1996).
31. Oltrogge, L. M. *et al.* Multivalent interactions between CsoS2 and Rubisco mediate α-carboxysome formation. *Nat Struct Mol Biol* **27**, 281–287 (2020).
32. Blikstad, C. *et al.* Identification of a carbonic anhydrase–Rubisco complex within the alpha-carboxysome. *Proceedings of the National Academy of Sciences* **120**, e2308600120 (2023).
33. Wang, H. *et al.* Rubisco condensate formation by CcmM in β-carboxysome biogenesis. *Nature* **566**, 131–135 (2019).
