# Supplementary material for: In-cell structure and variability of pyrenoid Rubisco
Source: Nat Commun. 2025 Aug 20;16:7763. doi: 10.1038/s41467-025-62998-y (PMC12368222; doi:10.1038/s41467-025-62998-y)
Supplement: Supplementary file 2 — Description of Additional Supplementary File [file 41467_2025_62998_MOESM2_ESM.pdf]

The Description of Additional Supplementary File

Supplementary Movie 1: A movie showing the slicing through a pyrenoid tomogram with the segmentation and Rubisco particles mapped back.
